# Identification of subclinical iron deficiency using new erythrocytes, leukocytes, and reticulocytes parameters during nonsevere acute infection in pediatric outpatients

**DOI:** 10.55730/1300-0144.5509

**Published:** 2022-05-29

**Authors:** Nazmi Mutlu KARAKAŞ, Serap KİRKİZ, Zühre KAYA

**Affiliations:** 1 Department of General Pediatric, Faculty of Medicine, Gazi University, Ankara, Turkey; 2 Department of Pediatric Hematology, Faculty of Medicine, Gazi University, Ankara, Turkey

**Keywords:** Iron deficiency, child, infection

## Abstract

**Background/aim:**

This study aims to investigate the diagnostic utility of new erythrocytes, leukocytes, and reticulocytes parameters for the identification of subclinical iron deficiency (ID) in children under 6 years with nonsevere acute infection in pediatric outpatients.

**Materials and methods:**

The study included 102 children with acute infections and 31 true ID. Traditional and new hematology parameters were measured in a Sysmex-XN^®^, along with C-reactive protein level, and iron parameters. Participants’ ID were categorized as: the ferritin < 100 ng/mL, transferrin saturation < 20% was defined as “subclinical or functional ID (FID) in Group 1”; ferritin < 30 ng/mL, transferrin saturation < 20%, as “absolute-ID (AID)” in Group 2; ferritin < 12 ng/mL without anemia and infection, as “true ID” in Group 3.

**Results:**

The frequencies of FID and AID among the 102 children with acute infection were 24% and 76%, respectively. Compared with the Group 2 patients, Group 1 had a significantly higher mean percentage of hypochromic erythrocytes (Hypo-He), and significantly lower levels of hemoglobin (Hb) and Hb content of reticulocytes (RET-He) (p < 0.05 for all). Compared with Group 2 and Group 3 patients, Group 1 had a significantly higher mean percentage of immature reticulocyte fraction (IRF) and immature granulocyte (IG) values (p < 0.05 for all). The RET-He, IRF%, Hypo-He%, and IG% cut-off values for predicting FID during infection were 27.0 pg, 10.6%, 2.5%, and 0.35% respectively.

**Conclusion:**

The RET-He, Hypo-He, IRF, and IG may be useful parameters for identifying subclinical ID in small children with nonsevere acute infection in pediatric outpatients.

## 1. Introduction

Iron deficiency (ID) and iron deficiency anemia (IDA) remain some of the major public health problems in the world today [1]. A comprehensive study has reported that the prevalence of ID and IDA in young children were 32.5% and 7.8%, respectively [2]. Early diagnosis of ID and the prompt initiation of iron therapy is primarily important to the prevention of IDA in children [3,4].

Complete blood counts (CBCs) are widely used as one of the most important tests for diagnosing acute infection and IDA [5,6]. Modern hematology analyzers can now reliably measure a broader panel of CBC parameters including reticulocyte hemoglobin content (RET-He) and hypochromic erythrocytes (Hypo-He), and these can help discriminate functional or subclinical iron deficiency (FID) from absolute iron deficiency (AID) in the inflammatory phase of chronic disease, but data are limited regarding the clinical utility of RET-He and Hypo-He values for identifying ID/IDA in children with acute inflammatory conditions such as acute infection [7–11].

In this study, we investigated the utility of new erythrocytes, leukocytes, and reticulocytes parameters for identifying subclinical ID in small children with nonsevere acute infections in pediatric outpatient settings (POSs).

## 2. Methods

This cross-sectional study was approved by the Gazi University Institutional Review Board. All parents gave informed consent. The participants were children between 6 months and 5 years of age who were assigned ICD 10 codes “B34.9,” or “A49.9,” or “R50.9” for common nonsevere infections and “E61.1” for ID upon admission to our POSs during the winter months. Patients with nonsevere acute infections were considered for enrollment if they met one or more inclusion criteria: i) presenting complaint of fever; ii) clinically apparent infection recorded as rhinosinusitis, acute otitis media, acute tonsillitis, or pneumonia; iii) streptococcal pharyngitis or urinary tract infection diagnosed by positive culture of throat or urine sample; iv) antibiotic therapy administered based on the clinician’s judgment. Healthy controls who were admitted for preoperative evaluation were included. The exclusion criteria were sepsis, anemia of chronic disease, immune thrombocytopenic purpura (ITP), malignancy, and hospitalization. Children who did not undergo iron status or CRP testing were also excluded.

Of the 543 total patients screened, 133 were enrolled. Hemogram with reticulocyte and conventional serum iron parameters (serum iron [SI], total iron-binding capacity [TIBC], and serum ferritin) and serum CRP level were tested. Patients were assigned to the FID group (Group 1) and AID group (Group 2) and true ID group (Group 3) according to hemogram, CRP level, iron status. As described previously, FID was defined as transferrin saturation < 20% and ferritin < 100 ng/mL in association with elevated CRP level, whereas AID was defined as transferrin saturation < 20% and ferritin < 30 ng/mL in association with normal CRP level during infection. True ID was defined as ferritin < 12 ng/mL without anemia (Hb >11gr/dL) and infection [1,4,12].

### 2.1. Hematological parameters and analyses

Serum iron level was photometrically measured on AU5800 analyzer (Beckman Coulter, USA). Serum ferritin was measured separately on the DXI800 analyzer (Beckman Coulter, USA). Total iron-binding capacity is a measure of plasma iron concentration as reflected by plasma transferrin. Transferrin saturation was calculated as SI divided by TIBC and expressed as a percentage. Serum CRP level was measured using a nephelometric assay (Beckman Instruments, California) with the normal range specified as 0 to 6 mg/L.

Routine CBC parameters including hemoglobin (Hb), mean corpuscular volume (MCV), mean corpuscular hemoglobin (MCH), mean corpuscular Hb concentration (MCHC), red blood cell (RBC) count, red cell distribution volume (RDW), reticulocyte (RET) count, leukocyte count, and counts and percentages of neutrophils, lymphocytes, and monocytes and new CBC parameters were studied in the Sysmex-XN^®^ 2000 (Sysmex Corporation, Kobe, Japan). Age- and gender-specific reference ranges for newer CBC parameters and reticulocyte indices evaluated are described below [13].

#### 2.1.1. Reticulocyte parameters

Immature reticulocyte fraction (IRF) was the total percentage of immature reticulocytes, calculated as the sum of medium-fluorescent reticulocyte percentage (MFR) plus high-fluorescent reticulocyte percentage (HFR). Reticulocyte production index (RPI) was automatically calculated as the following ratio: (reticulocyte percentage [RET%] (×) hematocrit [HCT]) divided by (0.45 (×) reticulocyte survival days in the blood), where 0.45 represents the ideal HCT. Other new reticulocyte-related parameters analyzed were Hb content of reticulocytes (RET-He in pg).

#### 2.1.2. Erythrocyte parameters

The new erythrocyte parameters measured were percentage hypochromic RBCs with Hb content < 17 pg (Hypo-He%); percentage hyperchromic RBCs with Hb content > 49 pg (Hyper-He%); percentage microcytic RBCs with volume < 60 fL (Micro-R%); percentage macrocytic RBCs with volume > 120 fL (Macro-R%).

#### 2.1.3. Leukocyte parameters

The new leukocyte parameters measured were immature granulocyte count (IG); high-fluorescent lymphocyte count (HFLC).

### 2.2. Statistical analysis

All data were analyzed using SPSS 15.0. Results were compared using the Mann-Whitney U test. Chi-square testing was applied for categorical data. Spearman correlation coefficient was used for the correlation analysis. Receiver operating characteristic (ROC) analysis was performed. P values <0.05 were considered statistically significant.

## 3. Results

The demographic and clinical characteristics of the 133 patients are summarized in Table 1. The frequencies of FID and AID among the 102 children with acute infection were 24% and 76%, respectively. The remaining 31 children were diagnosed with true ID. There were no significant differences between the groups with respect to age or sex (p > 0.05 for both).

### 3.1. Infection types and treatment

The most common primary sites in order of frequency were ear-nose-throat, urinary tract system, and lung in Table 1. Compared with the Group 2 patients, Group 1 had a significantly lower rate of ear-nose-throat infection, and significantly higher rates of streptococcal pharyngitis, and urinary tract infection (p < 0.05 for all). The mean values for serum ferritin, ESR, and CRP levels were significantly higher in Group 1 than in Group 2 and Group 3 (p < 0.05 for all). The rate of antibiotics in Group 1 was significantly higher than that in Group 2 (p < 0.05).

### 3.2. Comparison of erythrocyte and reticulocyte parameters

Data are summarized in Tables 2 and 3. Compared to Group 2, Group 1 had significantly lower mean values for serum Hb, RBC, and RET-He (p < 0.05 for both). Group 1 also had significantly higher mean values for Hypo-He than Group 2 (p < 0.05). Group 1 had significantly higher mean values for IRF, MFR, HFR compared to Group 2 and Group 3 (p < 0.05 for all).

### 3.3. Leukocytes parameters

Data are summarized in Table 4. Compared to Group 2 and Group 3, Group 1 had significantly higher mean values for monocyte count, HFLC, IG, monocyte%, HFLC%, and IG% (p < 0.05 for all). Compared to Group 3, Group 1 had significantly higher mean values for leukocyte count (p < 0.05 for all).

### 3.4. Correlation of leukocyte parameters and CRP level

The IG (%) (r = 0.72, p: 0.0001) and IG count (r = 0.68, p: 0.0001) were positive moderately correlated with CRP in Group 1.

### 3.5. Receiver operating characteristic analysis

**Figure 1a:**
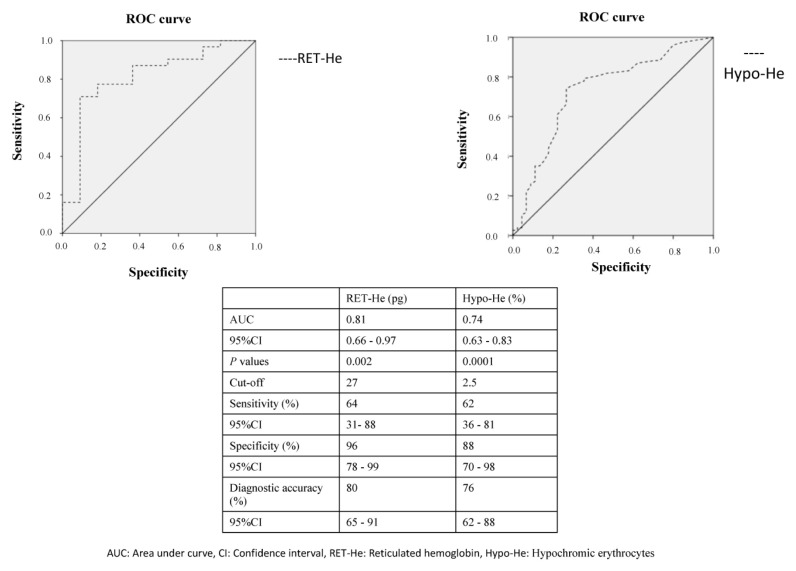
Hb content of reticulocytes (RET-He) index for identifying subclinical iron deficiency in children with acute infections. Figure 1b. Receiver operating characteristic curve analysis of hypochromic erythrocytes (Hypo-He). IG: immature granulocyte, AUC: area under curve, CI: confidence interval

**Figure 2:**
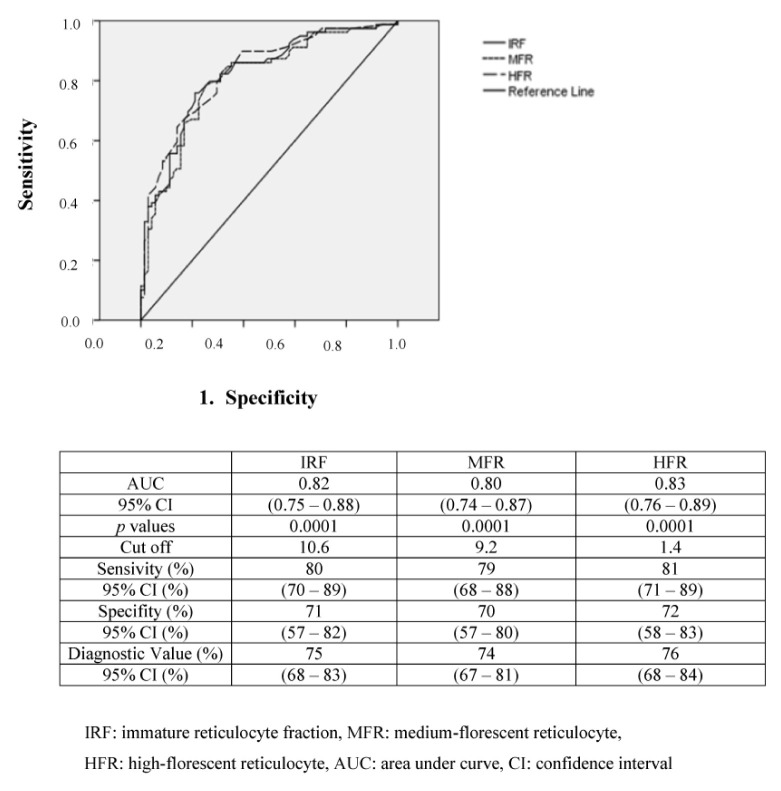
Receiver operating characteristic curve analysis of immature reticulocyte fraction (IRF), medium-fluorescent reticulocyte percentage (MFR) and high-fluorescent reticulocyte percentage (HFR) for identifying subclinical iron deficiency in children with acute infections. IRF: Immature reticulocyte fraction, MFR: Medium-florescent reticulocyte, HFR: High-florescent reticulocyte, AUC: Area under curve, CI: Confidence interval

**Figure 3:**
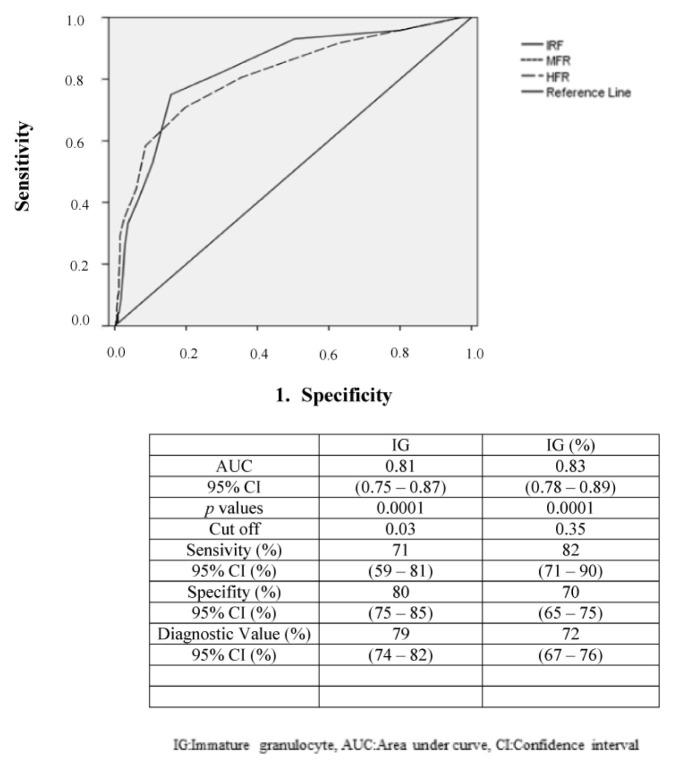
Receiver operating characteristic curve analysis of immature granulocyte parameters for identifying subclinical iron deficiency in children with acute infections. IG: Immature granulocyte, AUC: Area under curve, CI: Confidence interval

The results of ROC analysis for identifying subclinical ID or FID are shown in Figures 1a, 1b, 2 and 3. Regarding erythrocytes and reticulocyte parameters, the established area under the curve (AUC) cut-off values were 10.6% for IRF, 9.2% for MFR, 1.4% for HFR, 2.5% for Hypo-He, and 27.0 pg for RET-He. Sensitivity was highest for IRF (80%; 95% confidence interval [CI] 70%–89%) and HFR (81%; 95%CI 71%–89%) followed by MFR (79%; 95%CI 68%–88%) RET-He (64%; 95% CI 31%–88%) and Hypo-He (62%;95%CI 36%–81%). Specificity was highest for RET-He (96%; 95% confidence interval [CI] 78%–99%) and Hypo-He (88%; 95%CI 70%–98%) followed by IRF (71%; 95%CI 57%–82%) MFR (70%; 95% CI 57%–80%) and HFR (72%;95%CI 58%–83%). Regarding leukocyte parameters, the established area under the curve (AUC) cut-off values were 0.03 (x) 10^9^/L for IG, and 0.35% for IG%. Sensitivity was higher for IG% (82%; 95%CI 71%–90%) compared with IG (71%; 95%CI 59%–81%). Specificity was also higher for IG (80%; 95%CI 75%–85%) compared with IG% (70%; 95%CI 65%–75%).

## 4. Discussion

In this study, we found the subclinical ID or FID frequency of (24%) and the AID frequency of (76%) among 102 children less than 6 years old with nonsevere acute infection in POSs. Similarly, the literature indicates a wide range of FID incidence, from 13% to 90%, in children with the inflammatory phase of chronic conditions [14–16]. During the acute infection or the inflammatory phase of chronic disease, serum iron and ferritin are increased, whereas transferrin is decreased. This means that iron parameters are less reliable markers for diagnosing ID in these conditions. Serum transferrin receptor level and hepcidin may be useful for distinguishing anemia of inflammation from iron deficiency anemia (IDA), but these tests are not widely available [17]. Previous authors have documented the use of new red cell parameters in hematology analyzers for discriminating between FID and AID in the inflammatory phase of chronic disease [14–16]. Absolute and true ID have been well described in children less than 6 years old according to the World Health Organization guideline, but recognizing FID during acute infection can be challenging [1].

Functional iron deficiency is a major component of anemia of chronic disease [14–16]. It is most commonly identified as normocytic or normochromic anemia, but can become microcytic and hypochromic as the disease progresses. Reticulocytosis is not usually observed. The recent development of automated RET counts has allowed the more precise counting of these cells, as well as an objective measure of their maturity based on RNA content [7,18]. New reticulocyte and erythrocyte parameters, such as RET-He, RPI, IRF, Micro-R, and Hypo-He, are useful parameters for evaluating erythropoietic activity in FID [19]. Of these, RET-He (< 25pg) and the percentage of hypochromic red cells (≥ 6%) have been incorporated into the revised guidelines associated with anemia of chronic disease [12]. Similarly, in the initial phase of infection, ID can be mild to moderate, and if it goes unrecognized, the child may develop anemia. Using the RET-He and Hypo-He, physicians can identify ID before IDA develops. Reticulocyte Hb content is an early and sensitive marker of iron-restricted erythropoiesis in FID, whereas the proportion of hypochromic red cells is a time-averaged marker. Both these indices are similar in anemic patients, as are glucose and HbA1C in diabetic patients. In accord with these data, we identified subclinical ID with high specificity, moderate sensitivity, and diagnostic values of RET-He and Hypo-He in children with nonsevere acute infections. Advanced red cell parameters such as RET-He and Hypo-He are useful parameters for identifying subclinical ID in children with nonsevere acute infections because RET-He is not influenced by acute phase response [20].

Automated counting of reticulocytes enabled the introduction of the new reticulocyte parameter IRF [7,18]. This value is thought to be more sensitive than absolute or corrected RET counts for detecting recovery of bone marrow function. A finding of increased IRF during ID indicates that MFR and/or HFR is high. This suggests that IRF could potentially be an early diagnostic marker for IDA. Many researchers have reported data related to the clinical utility of IRF, MFR, and HFR in diagnosing and monitoring different types of anemia [7,21–23]. The IRF increases earlier than RET count, and therefore can be useful for monitoring the efficacy of therapy in IDA. In situations where erythropoiesis is ineffective, IRF is also increased while RET count is reduced or normal, as demonstrated in some cases with acute infections. In one study, the mean MFR and HFR in children with IDA were 10.3% and 2.3%, respectively [23]. Similarly, in our study, we identified cut-off values for predicting FID as 10.6% for IRF, 9.2% for MFR, and 1.4% for HFR. These values closely matched the means of 10.7% for IRF, 9.2% for MFR, and 1.5% for HFR that were reported for anemia of acute infection in children from Seoul, Korea [7]. Our findings indicate that these markers are moderately sensitive and specific markers for distinguishing FID from AID during infections in children who present POSs. However, the reliability of the IRF determination has been questioned because it is based on a very small number of counted cells. The gating for the HFR fraction is such that it is extremely susceptible to interference from other cellular blood elements, primarily leukocytes or large platelets. Some of these cells tend to enter the gating area selected for IRF determination, and will be falsely counted as part of the HFR value [18]. Therefore, we excluded patients with sepsis, ITP, and malignancy to prevent interference with large platelet and high leukocyte counts. Regardless, IRF is a promising parameter that needs to be part of assessments in clinical practice, especially acute infections.

The earliest elevated marker during infection is CRP [24]. As an acute-phase protein, raised CRP level is often a useful sign of an acute infection or the acute exacerbation of chronic disease [14,25]. Thus, the rapid availability of CBC and CRP results could provide a considerable advantage for diagnosing children with acute infections. We observed higher mean values for HFLC and IG values in the FID Group. Several observations have been reported for severe bacterial infections in children, while a relationship between IG and nonsevere infections has only been reported in only one comprehensive study of children in POSs as confirmed here [8,26,27]. This study suggested that the threshold 0.04 (x) 10^9^/L for IG and 0.3% in children younger than 10 years could be useful tools for identifying common acute infections in POSs [7]. Similarly, we found IG and IG% to be highly sensitive and specific for predicting FID in children less than 6 years old. Our cut-off levels were 0.03 (x) 10^9^/L for IG and 0.35% for IG%, respectively. In addition, statistically significant, moderately sized correlations were demonstrated between CRP level and IG values in the present study. Our results suggest that similar to CRP, IG could be a useful adjunctive marker for identifying FID in children with acute infection.

Several inflammatory markers, including leukocyte populations and validated CRP levels, should enable physicians to reliably identify children who need antibiotic treatment and help avoid treating those with viral disease [24,25]. One study showed that the antibiotic prescription rate was 56% higher for children with elevated CRP compared to those with normal CRP levels, thus this approach was indicated to prevent unnecessary antibiotic use in POS [24]. Another study revealed that of all febrile children with elevated CRP who were prescribed antibiotics in a POS, only 62% actually had bacterial infections [25]. In our study, we observed a similar antibiotic prescription rate (58%) based on a clinical and laboratory finding of elevated CRP, increased IG counts, and positive culture in febrile pediatric outpatients.

The first limitation of this study was that iron status and hemostatic testing were not repeated after recovery to confirm whether ID was restricted to the infection period. The second limitation was that all nonculture and culture-based tests were not performed to distinguish between viral and bacterial infections in POSs. The third limitation was the small sample size.

Our data indicate that RET-He, Hypo-He, IRF, and IG in addition to conventional iron parameters may be useful parameters for the early detection of subtle ID in children under 6 years with nonsevere acute infections at POS.

## Figures and Tables

**Table 1:** Demographic, clinical and laboratory characteristics of the 133 pediatric outpatients with/without nonsevere acute infections.

Variable	Group 1 n: 24	Group 2 n: 78	Group 3 n: 31	P1	P2	P3
Age (yrs)	2.6 ± 1.7	3.2 ± 1.5	2.9 ± 1.4	0.12	0.36	0.42
Males/Females	13 (55%)/11 (45%)	42 (54%)/36 (46%)	15 (48%)/16 (52%)	0.98	0.67	0.76
**Clinically documented infections**						
Ear-nose-throat (otitis, tonsilitis, rhinosinusitis)	14 (58%)	71 (91%)	-	**0.004**	**-**	**-**
Pneumonia	1 (4%)	2 (3%)	-	0.88	**-**	**-**
**Microbiologically documented infections**						
Throat culture (AGBHS)	5 (22%)	2 (2%)	-	**0.007**	-	-
Urine culture (100,000 colonies *E. coli, K*lebsiella)	4 (16%)	3 (4%)	-	**0.04**	-	-
**Laboratory parameters**						
ESR (mm/h)	44.6 ± 6.2	15.1 ± 5.5	11.1 ± 1.7	**0.002**	**0.001**	0.15
CRP (mg/L)	48.5 ± 11.7	2.1 ± 0.1	1.1 ± 0.1	**0.009**	**0.0001**	0.96
Serum iron (μg/dL**)**	56.3 ± 8.1	64.4 ± 5.1	39.8 ± 9.	0.70	0.28	0.12
SIBC (μg/dL**)**	310.9 ± 62.8	313.8 ± 53.1	362.8 ± 32.1	0.96	0.08	0.16
Ferritin (ng/mL)	74.3 ± 28.1	17.2 ± 4.9	8.2 ± 2.6	**0.002**	**0.0001**	**0.01**
**Treatment**						
Antibiotic administered	14 (58%)	22 (25%)	-	**0.005**	**-**	**-**

Group 1: FID: functional iron deficiency; Group 2: AID: absolute iron deficiency; Group 3: True iron deficiency AGBHS: A group beta-hemolytic *Streptococcus*, ESR: Erythrocyte sedimentation rate, CRP: C-reactive protein, SIBC: Serum iron-binding capacity. p_1_: Group 1 vs. Group 2, p_2_: Group 1 vs. Group 3, p_3_: Group 2 vs. Group 3

**Table 2:** Comparison of erythrocytes parameters in the three groups of pediatric patients with/without acute infections.

Items	Group 1 (n:24)	Group 2 (n:78)	Group 3 (n:31)	P1	P2	P3
Hb (g/dl)	11.7 ± 0.3	12.5 ± 0.7	12.2 ± 0.4	**0.001**	0.07	0.25
RBC (10^12^/L)	4.6 ± 0.5	4.9 ± 0.4	4.8 ± 0.3	**0.01**	0.07	0.15
MCV (fl)	76.9 ± 6.4	77.3 ± 4.5	75.1 ± 6.9	0.09	0.79	0.06
MCH (pg)	26.5 ± 3.1	25.6 ± 2.2	25.4 ± 3.2	0.66	0.76	0.87
MCHC (g/dL)	32.5 ± 2.1	33.1 ± 1.3	32.7 ± 1.8	0.10	0.45	0.43
RDW (%)	14.7 ± 2.6	13.5 ± 2.1	14.4 ± 2.4	0.21	0.93	0.11
Micro-R (%)	16.7 ± 3.4	12.3 ± 1.2	18.5 ± 4.4	0.08	0.81	0.06
Macro-R (%)	3.5 ± 0.9	3.9 ± 0.5	3.6 ± 0.3	0.14	0.35	0.19
Hypo-He (%)	15.6 ± 4.6	4.4 ± 2.3	5.8 ± 3.2	**0.04**	0.23	0.35
Hyper-He (%)	0.3 ± 0.09	0.5 ± 0.1	0.3 ± 0.08	0.07	0.95	0.09

Micro-R: Microcytic-R, Macro-R: Macrocytic-R, Hypo-He: Hypochromic hemoglobin, Hyper-

Group 1: FID: Functional iron deficiency; Group 2: AID: Absolute iron deficiency; Group 3: True iron deficiency.

p_1_: Group 1 vs. Group 2, p_2_: Group 1 vs. Group 3, p_3_: Group 2 vs. Group 3

**Table 3:** Comparison of reticulocytes parameters in the three groups of pediatric patients with/without acute infections.

Items	Group 1 (n:24)	Group 2 (n:78)	Group 3 (n:31)	P1	P2	P3
RET-He	23.5 ± 2.8	29.1 ± 0.9	28.8 ± 1.9	**0.02**	0.17	0.77
RET (10^12^/L)	52.7 ± 9.7	54.7 ± 4.6	52.4 ± 9.1	0.86	0.81	0.08
RET (%)	1.1 ± 0.3	1.2 ± 0.4	1.0 ± 0.2	0.88	0.15	0.09
RPI (%)	0.5 ± 0.1	0.7 ± 0.3	0.5 ± 0.2	0.26	0.53	0.20
IRF (%)	15.8 ± 1.2	11.1 ± 1.3	9.1 ± 1.6	**0.02**	**0.01**	0.45
MFR (%)	12.1 ± 1.1	8.9 ± 0.7	8.1 ± 3.1	**0.03**	**0.02**	0.64
HFR (%)	3.9 ± 1.0	2.2 ± 0.6	0.9 ± 0.3	**0.04**	**0.03**	0.40

IRF: Immature reticulocyte fraction; MFR: Medium-fluorescent reticulocyte percentage; HFR: High fluorescent reticulocyte percentage, RPI: Reticulocyte production index, RET-He: Hemoglobin content of the reticulocytes,

Group 1: FID: Functional iron deficiency; Group 2: AID: Absolute iron deficiency; Group 3: True iron deficiency.

p_1_: Group 1 vs. Group 2, p_2_: Group 1 vs. Group 3, p_3_: Group 2 vs. Group 3

**Table 4:** Comparison of leukocytes parameters in the three groups of pediatric patients with/without acute infections.

Items	Group 1 (n:24)	Group 2 (n:78)	Group 3 (n:31)	P1	P2	P3
Leukocyte (10^9^/L)	9.8 ± 3.3	8.9 ± 2.7	8.1 ± 1.9	0.20	**0.04**	0.27
Neutrophil (%)	37.9 ± 19.7	37.2 ± 13.9	34.3 ± 10.5	0.86	0.67	0.24
Neutrophil (10^9^/L)	4.1 ± 2.8	3.4 ± 1.7	2.7 ± 1.1	0.78	0.21	0.11
Lymphocyte (%)	49.5 ± 18.2	50.5 ± 13.6	54.8 ± 10.4	0.92	0.45	0.14
Lymphocyte (10^9^/L)	4.6 ± 2.2	4.4 ± 1.7	4.5 ± 1.5	0.88	0.77	0.82
Monocyte (%)	10.6 ± 5.5	7.9 ± 3.3	8.1 ± 2.4	**0.03**	**0.04**	**0.28**
Monocyte (10^9^/L)	1.0 ± 0.5	0.7 ± 0.3	0.6 ± 0.2	**0.005**	**0.007**	**0.82**
IG (%)	0.4 ± 0.03	0.3 ± 0.02	0.2 ± 0.03	**0.04**	**0.03**	**0.44**
IG (10^9^/L)	0.04 ± 0.006	0.03 ± 0.002	0.02 ± 0.002	**0.03**	**0.01**	**0.26**
HFLC (%)	1.1 ± 0.1	0.5 ± 0.03	0.4 ± 0.03	**0.003**	**0.002**	**0.68**
HFLC (10^9^/L)	0.09 ± 0.008	0.04 ± 0.002	0.03 ± 0.002	**0.001**	**0.001**	**0.37**

He: Hyperchromic hemoglobin, IG: Immature granulocyte,

Group 1: FID: Functional iron deficiency; Group 2: AID: Absolute iron deficiency; Group 3: True iron deficiency.

p_1_: Group 1 vs. Group 2, p_2_: Group 1 vs. Group 3, p_3_: Group 2 vs. Group 3
